# Diagnostic accuracy of serum alanine aminotransferase as biomarker for nonalcoholic fatty liver disease and insulin resistance in healthy subjects, using 3T MR spectroscopy

**DOI:** 10.1097/MD.0000000000006770

**Published:** 2017-04-28

**Authors:** Jose Luis Martin-Rodriguez, Jorge Gonzalez-Cantero, Alvaro Gonzalez-Cantero, Juan Pedro Arrebola, Jorge Luis Gonzalez-Calvin

**Affiliations:** a Department of Radiology, University Hospital San Cecilio; b Department of Radiology, HGU Gregorio Marañón Madrid Spain, and University of Granada; c Complejo Hospitalario de Toledo, Toledo, Castilla-La Mancha; d Complejo Hospitalario Universitario de Granada, Instituto de Investigación, Biosanitariaibs CIBERESP; e Department of Gastroenterology, University Hospital San Cecilio, University of Granada, Granada, Spain.

**Keywords:** insulin resistance, magnetic resonance spectroscopy, NAFLD, sensitivity and specificity

## Abstract

Recognition of the close relationship of nonalcoholic fatty liver disease (NAFLD) with diabetes mellitus 2, obesity, metabolic syndrome, and cardiovascular disease has stimulated growing interest in NAFLD as a public health problem. Serum alanine aminotransferase (ALT) has been proposed as a marker of NAFLD, but levels are within the range currently considered “normal” in a large proportion of NAFLD subjects.

The aim of the study was to determine the diagnostic accuracy of serum ALT for identifying individuals with NAFLD, using 3-Tesla (T) magnetic resonance spectroscopy (^1^H-MRS).

A cross-sectional study was conducted in 129 healthy subjects. Liver triglyceride content was quantified by ^1^H-MRS. NAFLD was defined as liver triglyceride content greater than 5.56%.

Liver triglyceride content was >5.56% in 79 participants (NAFLD) and lower in the remaining 50 (normal). Serum ALT levels correlated positively with liver triglyceride content (*r* = 0.58, *P* < .001), Homeostatic Model Assessment for Insulin Resistance (*r* = 0.32, *P* < .01), and fasting insulin (*r* = 0.31, *P* < .01), and inversely correlated with adiponectin (*r* = 0.35, *P* < .01) and high-density lipoprotein cholesterol (*r* = 0.32, *P* < .01). Regression analysis showed that serum ALT was the best predictor of NAFLD (*P* < .01). Optimal serum ALT cut-off to predict NAFLD was 23 IU/L (area under receiver-operating characteristic curve: 0.93; sensitivity: 0.94; specificity: 0.72).

This study shows that serum ALT is a sensitive and accurate biomarker of NAFLD if the “normal” ALT value is revised and established at a lower level. An ALT threshold of 23 IU/L identified 94% of individuals with NAFLD in the present series, using 3-T ^1^H-MRS for liver triglyceride quantification.

## 1 Introduction

Nonalcoholic fatty liver disease (NAFLD) is defined as fat accumulation in the liver in the absence of significant alcohol consumption or any other etiology for secondary hepatic steatosis. It is considered the hepatic manifestation of the metabolic syndrome (MetS), and is the most frequent cause of aminotransferase elevation.^[1]^ NAFLD encompasses simple hepatic steatosis, that is, the accumulation of triglycerides in the liver with no evidence of hepatocyte injury or inflammation, and nonalcoholic steatohepatitis, that is, the presence of hepatic steatosis with hepatocellular injury and inflammation that may eventually progress to liver cirrhosis and hepatocellular carcinoma.^[1,2]^

The prevalence of NAFLD varies widely from 10% to 52% in the general population, and up to 60% to 90% in high-risk persons with obesity, type 2 diabetes (T2DM), and/or MetS.^[1,3–7]^ Estimates from liver biopsy series indicate that the prevalence of nonalcoholic steatohepatitis is much lower, ranging from 2% to 5%.^[5]^ Studies on the long-term outcome of patients with NAFLD showed that they more frequently die from cardiovascular disease (CVD) than from liver cirrhosis or hepatocellular carcinoma.^[6,8]^

Recognition of the close relationship of NAFLD with obesity,^[9]^ T2DM,^[10]^ MetS,^[11,12]^ insulin resistance,^[13]^ and CVD^[14–16]^ has stimulated growing interest in NAFLD as a public health problem, and in the search for a marker to identify individuals with NAFLD in the general population. After the demonstration by epidemiological and clinical studies of a frequent association between increased liver fat content and serum alanine aminotransferase (ALT) elevation, serum ALT has been proposed as a surrogate marker for NAFLD.^[17]^ Nevertheless, only a proportion of patients with NAFLD have elevated serum ALT.^[4,13,17–20]^

Given that serum ALT values within the current “normal” range have been associated with NAFLD and a higher risk of cardiometabolic disorders, persons with these conditions are included in the apparently healthy sample population that is considered “normal.” Therefore, it has been suggested that the upper “normal” limit for serum ALT should be re-evaluated to facilitate the identification of individuals with NAFLD.^[17,21]^ Prati et al, in a retrospective cohort study, proposed decreasing the upper limit of normal for serum ALT levels to ≤30 IU/L in men and ≤19 IU/L in women to detect more people with hepatitis C viremia. They used liver biopsies in 133 hepatitis C virus (HCV) antibody-positive persons, and ultrasound examination in 59 HCV antibody-negative blood donors,^[22]^ but no consensus has been reached.^[23]^ Recent cross-sectional studies are available on the sensitivity and specificity of serum ALT as a biomarker of NAFLD.^[16,24–26]^ However, in these studies, the NAFLD diagnosis was exclusively based on ultrasound imaging, but was not confirmed by liver biopsy or proton magnetic resonance spectroscopy (^1^H-MRS), which is the noninvasive gold standard for the quantification of liver triglyceride content,^[27,28]^ widely validated in clinical and epidemiological studies.^[13,29,30]^ Liver ultrasound examination is not sufficiently sensitive in cases of mild and moderate steatosis, and is likely to underestimate the prevalence of NAFLD.^[29,31,32]^

Thus, currently, the true diagnostic accuracy of serum ALT as biomarker for NAFLD in healthy subjects and its relationship with insulin resistance and other metabolic risk factors remain unknown. Clarification of this aspect may help to identify individuals with hepatic steatosis who might be more at risk of cardiometabolic diseases.

We have found no published study on the diagnostic accuracy, sensitivity, and specificity of serum ALT to identify individuals with NAFLD using 3-Tesla (T) ^1^H-MRS. We therefore utilized 3T (^1^H-MRS) to quantify liver triglyceride content in a sample of healthy adults to assess the accuracy, sensitivity, and specificity of serum ALT as a biomarker of NAFLD. We also investigated the relationship of liver triglyceride content and serum ALT with metabolic risk factors, including insulin resistance (Homeostatic Model Assessment for Insulin Resistance [HOMA-IR]), fasting insulin, adiponectin, and tumor necrosis factor (TNF), among others.

## 2 Material and methods

### 2.1 Study population

The study population was consecutively recruited between February 2011 and October 2013 from among individuals undergoing examination at the Occupational Risk Prevention Unit in Granada (Southern Spain) for a routine annual general checkup. Participants were all healthy male or female Caucasians aged between 19 and 76 years.

**Figure 1:**
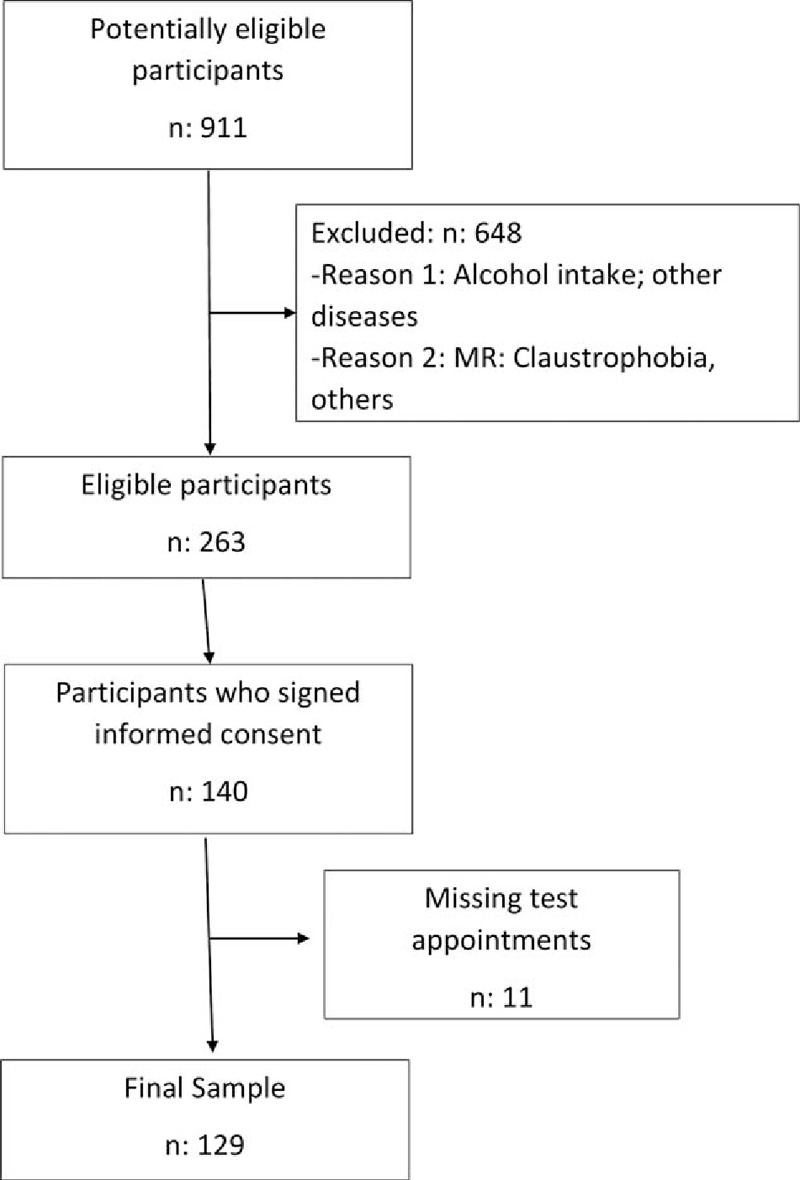
Flow of participants through the study.

Study exclusion criteria were: history of daily alcohol intake >20 g (men) or >10 g (women), based on responses to a validated questionnaire on alcohol consumption and confirmation of results by a family member; the presence of hepatitis B virus (HBV)/HCV serologic markers, autoimmune hepatitis, primary biliary cirrhosis, hemochromatosis, Wilson disease, cancer, diabetes mellitus, endocrine, cardiac, renal, or lung disease; the consumption of drugs that may cause steatosis (eg, corticosteroids, amiodarone, methotrexate, tamoxifen); body mass index (BMI) <17 or >40 kg/m^2^; and the wearing of a pacemaker or other device and/or self-reported claustrophobia incompatible with ^1^H-MRS. Out of the total 911 adults who came for the routine annual checkup, 263 individuals met the eligibility criteria, and 140 (53%) signed informed consent to participate in the study. After the exclusion of 11 of these individuals for missing test appointments, the final sample comprised 129 subjects with a mean age 45.7 years (range 20–76 years) (Fig. 1). The study was approved by the ethical committee of the University Hospital San Cecilio.

### 2.2 Study design and anthropometric evaluations

All individuals recruited for the study had undergone a full medical history, physical examination, complete blood analysis, and ultrasound examination as part of the screening process. The weight and height of the study participants were also recorded, calculating their BMI (kg/m^2^), and their waist circumference was measured with soft tape midway between the lowest rib and the iliac crest in standing position.

### 2.3 Laboratory analysis

Blood was drawn in the morning after overnight fasting. Serum ALT and aspartate aminotransferase (AST) levels were determined using a kinetic method (Cobas c 311, Roche Diagnostics GmbH, Mannheim, Germany), with coefficients of variation of 3.3 and 3.1, respectively; serum glucose by the glucose oxidase (enzymatic) method (Roche/Hitachi Analytics systems, Roche Diagnostics GmbH); adiponectin by radioimmunoassay (Linco Research, St. Charles, MO); serum insulin by electrochemiluminescence immunoassay (Elecsys 2010, Roche Diagnostics GmbH); serum TNF-α by enzyme-linked immunosorbent assay, using a TNF-α (human) ELISA kit (Biosource Europe, Nivelles, Belgium); and serum cholesterol with an enzymatic method (Roche Diagnostics GmbH). Insulin resistance was calculated as HOMA-IR = fasting insulin (mU/L) × fasting glucose (mmol/L)/22.5.^[33]^ Coefficients of variation of the biochemical tests ranged from 3.1% to 9.9%.

### 2.4 ^1^H-MRS 3T analyses

A magnetic resonance imaging study was conducted before the spectroscopy, acquiring in vivo spectra at 3T with a Philips Achieva system (Royal Philips, Amsterdam, Netherlands). A 3-plane localizer was employed to plan the ^1^H-MRS, and the spectra were obtained using the body coil of the scanner. Breath-hold was monitored using a respiratory belt.

A single voxel of 27 cm^3^ (30 × 30 × 30 mm) was selected within normal liver tissue in segment VI, avoiding the edge of the liver, the diaphragm, and major blood vessels. All spectra were obtained with a stimulated echo acquisition mode sequence (STEAM), setting the following parameters: repetition time = 8000; echo time = 20, 40, and 60 ms; number of signal averages = 4 (without water suppression); and bandwidth = 2000. Data were acquired within a breath hold. T2 correction was applied and field homogeneity was adjusted automatically for each voxel.

The MRS was reconstructed using Extended MR WorkSpace software (Philips). Raw data were zero-filled once, with no filter, and were phase-corrected, Fourier-transformed, baseline-corrected, and averaged. A Marquardt curve was fitted, using a combined Lorentzian–Gaussian model to calculate the area under the curve of fat and water peaks. Spectra were referenced to residual water and the dominant methylene lipid (–CH_2_) peak at δ = 4.47 and δ = 1.43 ppm, respectively. Fat fraction percentage (FF) was defined as FA/(FA + WA) × 100, where FA is the area under the fat peak and WA is the area under the water peak. ^1^H-MRS data were interpreted by an experienced radiologist blinded to the biochemical results.

Nonalcoholic fatty liver disease was defined by a liver fat content greater than 5.56%, as proposed in previous studies, and was classified as mild (>5.56% to 25% liver fat content), moderate (>25% to 50%), or severe (>50%).^[27,34]^

The anthropometric, biochemical, and ^1^H-MRS measurements of each individual were performed within a 24-hour period.

### 2.5 Statistical analysis

Results were expressed as means ± standard deviation (SD). The Kolmogorov–Smirnoff test was used to check the normality of the data distribution. Mean values were compared among groups with the 1-way analysis of variance (ANOVA), followed by the Tukey multiple-comparison test, the unpaired Student 2-tailed *t* test, or nonparametric Mann–Whitney *U* test, as appropriate. Correlations were examined by Pearson standard linear regression analysis (normal distribution) or by the Spearman test (non-normal distribution).

Backward stepwise multiple regression analysis was used to establish the most significant determinants of NAFLD. Variables entered into the equation were WC, BMI, HOMA-IR, and serum values of ALT, AST, GGT, fasting insulin, triglycerides, adiponectin, and high-density lipoprotein (HDL)-cholesterol. Only variables showing a *P* < .5 were retained in the final regression model.

Receiver-operating characteristic (ROC) curves were created, estimating optimal cut-off points for the diagnosis of NAFLD, and the sensitivity, specificity, positive predictive value (PPV), and negative predictive value (NPV) were calculated.

All analyses were performed with SPSS software for Windows, version 22 (SPSS Inc., Chicago, IL).

## 3 Results

The final study comprised 129 participants with a mean ± SD age of 45.7 ± 10.2 (range 20–76 years). Their anthropometric data are exhibited in Table 1 and their biochemical results are shown in Table 2. Seventy-five (58.13%) of the participants were diagnosed with NAFLD, classified as mild (29 cases), moderate (34 cases), or severe (12 cases); the remaining 54 individuals had liver fat content below 5.56%.

**Table 1:**
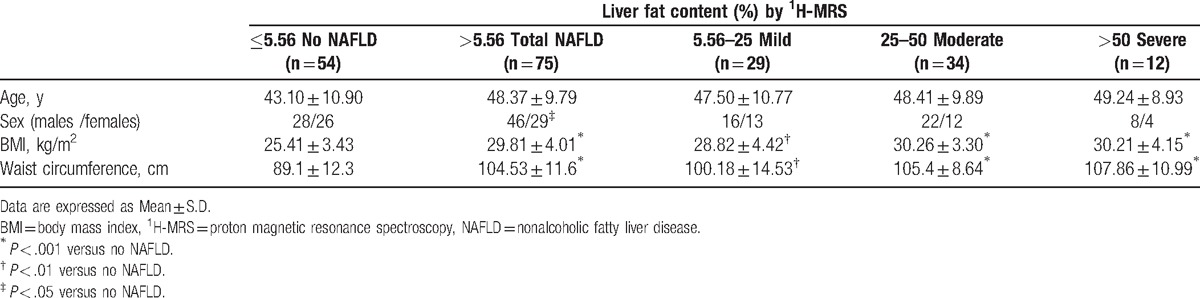
Anthropometric variables in subjects classified according to liver fat content.

**Table 2:**
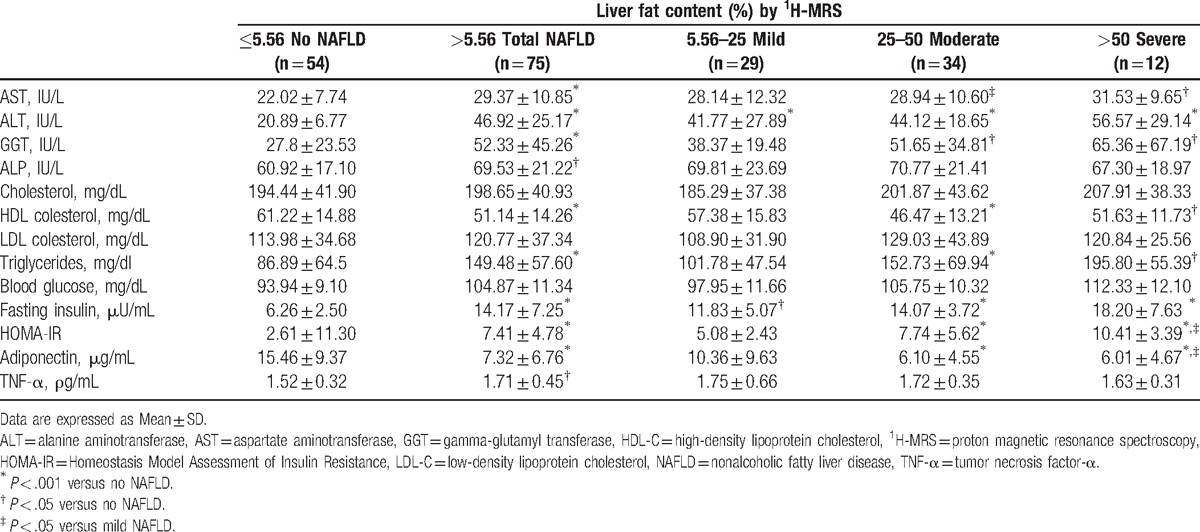
Biochemical variables in subjects classified according to liver fat content quantified by ^1^H-MRS.

### 3.1 Anthropometric and biochemical parameters according to liver fat content

As shown in Tables 1 and 2, mean BMI, waist circumference, HOMA-IR, serum ALT, AST, gamma-glutamyl transferase (GGT), alkaline phosphatase (ALP), triglycerides, TNF-α, and fasting insulin levels were higher in individuals with NAFLD than in those without (*P* < .001, except for ALP and TNF-α, *P* < .05); only HDL-cholesterol and adiponectin were lower in individuals with NAFLD than in those without (*P* < .001 for both). Multiple comparison tests among NAFLD categories only revealed significant differences in ALT (*P* < .05), HOMA-IR (*P* < .05), and adiponectin (*P* < .05) between individuals with severe versus mild steatosis.

### 3.2 Correlations of liver fat content with different parameters

Liver fat content was highly significantly and positively correlated with serum ALT (*r* = 0.58, *P* < .001) (Fig. 1), serum AST (*r* = 0.32, *P* < .01), serum GGT (*r* = 0.31, *P* < .01), waist circumference (*r* = 0.54, *P* < .001), BMI (*r* = 0.48, *P* < .001), fasting insulin (*r* = 0.57, *P* < .001), HOMA-IR (*r* = 0.57, *P* < .001), and serum triglyceride (*r* = 0.35, *P* < .01), and was inversely correlated with serum adiponectin (*r* = −0.43, *P* < .001) and HDL-cholesterol (*r* = −0.32, *P* < .01).

### 3.3 Correlations of serum ALT values with different parameters

**Figure 2:**
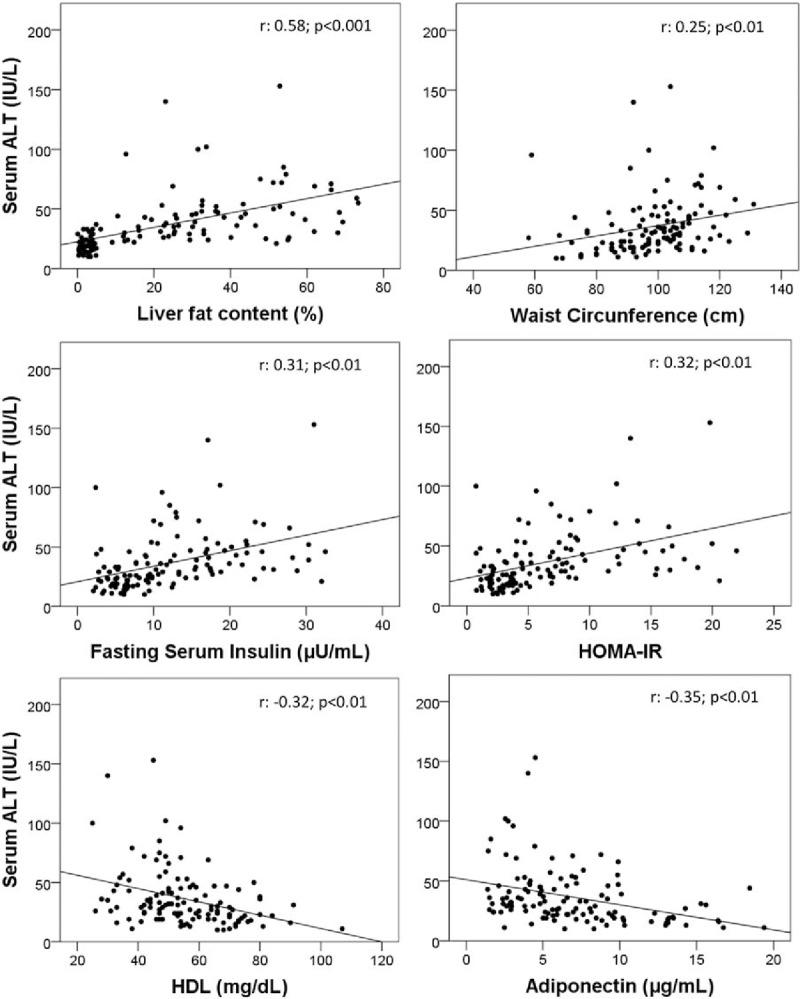
Correlation of serum ALT with: liver fat content, waist circumference, HOMA-IR, and serum levels of fasting insulin, adiponectin, and HDL-cholesterol. ALT = alanine aminotransferase, HDL = high-density lipoprotein, HOMA-IR = Homeostatic Model Assessment for Insulin Resistance.

The results depicted in Fig. 2 confirm that serum ALT values were positively correlated with liver fat content. Serum ALT was also correlated with HOMA-IR (*r* = 0.32, *P* < .01), serum fasting insulin (*r* = 0.31, *P* < .01), triglycerides (*r* = 0.18, *P* < .05), waist circumference (*r* = 0.25, *P* < .01), and BMI (*r* = 0.25, *P* < .01), and inversely correlated with adiponectin (*r* = −0.35, *P* < .01) and with HDL-cholesterol (*r* = −0.32, *P* < .01).

### 3.4 Regression analyses

Results of the backward stepwise regression analyses on the predictors of liver fat content showed that serum ALT was the most significant independent variable (β coefficient = 1.367, SE = 0.121, *P* < .01). HOMA-IR was also significant (β coefficient = 1.160, SE = 0.075, *P* < .04).

### 3.5 ROC curves and validity of serum ALT for the diagnosis of NAFLD

**Figure 3:**
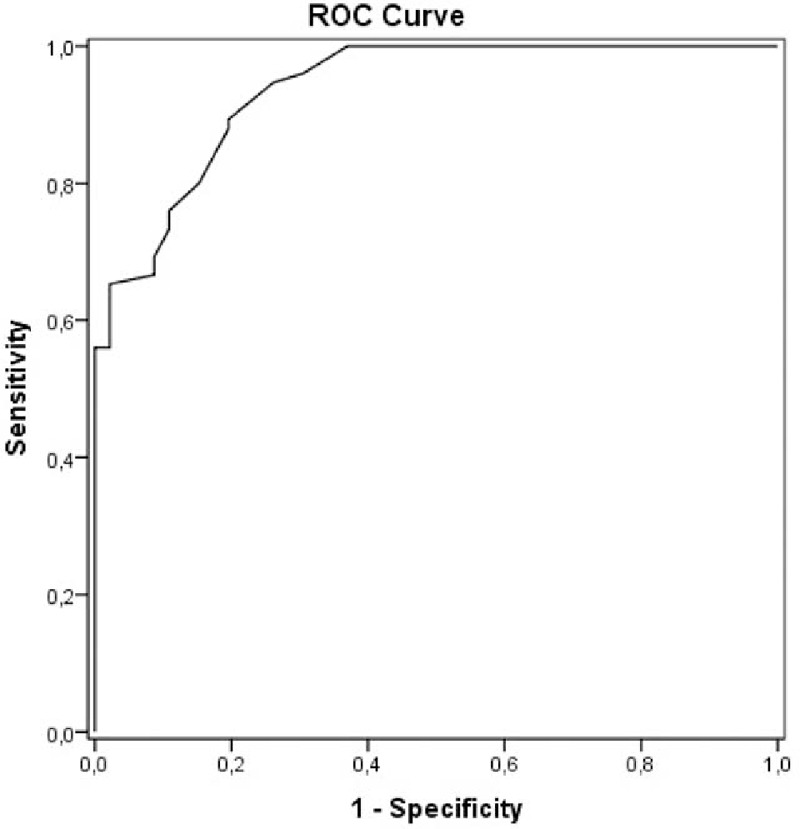
Receiver-operating characteristic curve of sensitivity plotted against 1-specificity of serum ALT to identify subjects with liver fat content greater than 5.56% quantified by proton magnetic resonance spectroscopy (^1^H-MRS) 3T. ALT = alanine aminotransferase, ^1^H-MRS = proton magnetic resonance spectroscopy.

**Figure 4:**
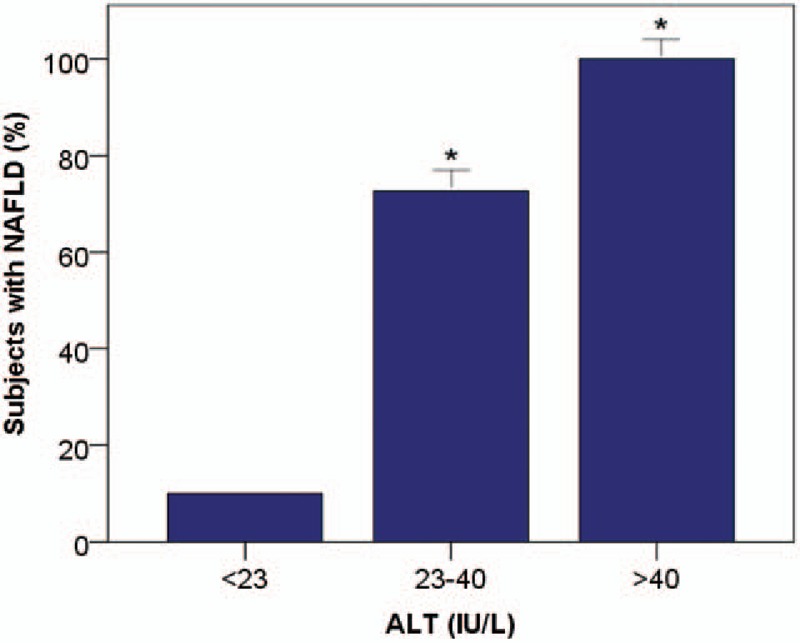
Percentage of subjects with NAFLD in the different ALT categories. ^∗^*P* < .001 versus no NAFLD. ALT = alanine aminotransferase, NAFLD = nonalcoholic fatty liver disease.

Receiver-operating characteristic (ROC) curves were created to assess the accuracy of serum ALT to predict liver fat content greater than 5.56% (upper limit of normal range) (Fig. 3). The area under the curve (AUC) was 0.93 (95% confidence interval [CI] 0.89, 0.97), the optimal ALT cut-off point was 23 IU/L, with a sensitivity of 0.94, specificity of 0.72, PPV of 0.82, and NPV of 0.90. Analysis by sex showed an optimal ALT cut-off value to identify individuals with NAFLD of 24 IU/L for the males (AUC: 0.92; sensitivity: 0.95; specificity: 0.67) and 21 IU/L for the females (AUC: 0.94; sensitivity: 0.96; specificity: 0.76). Figure 4 shows that 48% of individuals with ALT below 40 IU/L had NAFLD, and had significantly higher values of HOMA-IR and serum fasting insulin and lower values of adiponectin and HDL-cholesterol levels in comparison with those without NAFLD. Only 4 individuals with ALT levels below 23 IU/L had NAFLD. The AUC for the rest of the parameters had lower values: BMI: 0.82, waist circumference: 0.84, GGT: 0.74, and AST: 0.76.

### 3.6 Metabolic variables according to both liver fat content and ALT categories

**Figure 5:**
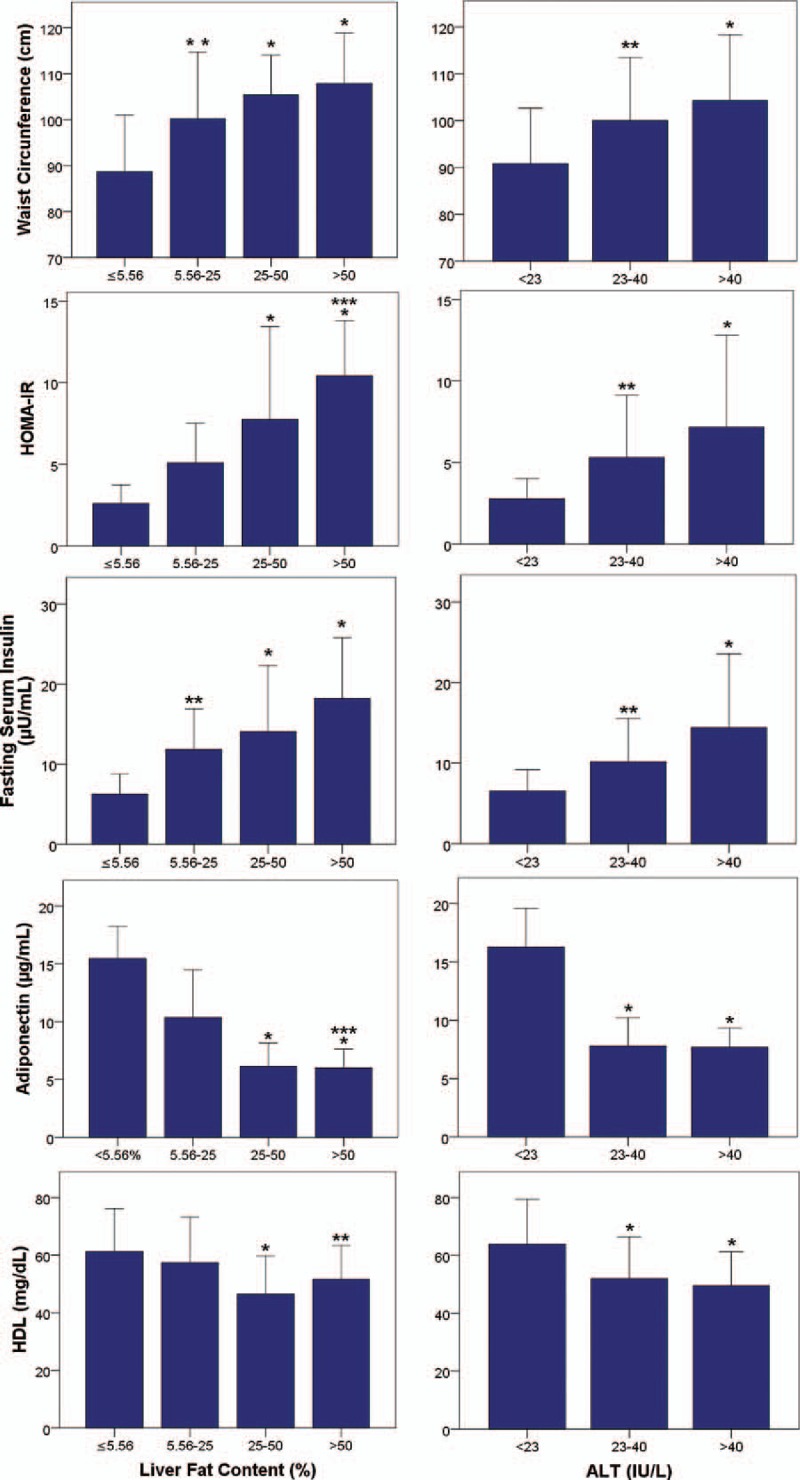
Metabolic variables changes according to both liver fat content and ALT categories. Data are presented as means ± SD. ^∗^*P* < .001 versus no NAFLD; ^†^*P* < .01 versus no NAFLD; ^‡^*P* < .05 versus mild NAFLD. ALT = alanine aminotransferase, NAFLD = nonalcoholic fatty liver disease.

As shown in Fig. 5, changes in waist circumference, HOMA-IR, and serum values of fasting insulin, adiponectin, triglycerides, and HDL-cholesterol in the different NAFLD categories paralleled changes in the different ALT categories.

## 4 Discussion

In this sample of individuals from the general population, serum ALT levels were strongly correlated with liver fat content and enabled the detection of most of the participants with NAFLD. Liver triglyceride content was determined by ^1^H-MRS, the only noninvasive reference method for its quantification,^[34–36]^ widely validated in population-based studies^[27]^ and clinical trials.^[37]^

The participants with NAFLD had higher serum levels of ALT, HOMA-IR, fasting insulin, and triglycerides, and lower levels of adiponectin and HDL-cholesterol in comparison with those without NAFLD, as previously reported in selected populations of obese or diabetic patients.^[13,38–40]^ Significant differences in ALT levels were found between subjects with mild NAFLD and those without NAFLD, and between those with severe and mild NAFLD, suggesting that serum ALT levels might offer the capacity to discriminate among different degrees of liver fat content.

Both serum ALT and liver fat content were significantly correlated with HOMA-IR, serum fasting insulin, and triglycerides, and were significantly inversely correlated with serum adiponectin and HDL-cholesterol. Parallelism observed between changes in these metabolic parameters in the different NAFLD categories and changes in the different ALT categories support the possible relationship between both NAFLD and serum ALT with insulin resistance and other metabolic risk factors. The present findings in healthy individuals are in agreement with results obtained in selected populations of obese individuals, and patients with diabetes or MetS.^[17–20,41]^

Serum adiponectin levels were inversely correlated with liver fat content, serum ALT, HOMA-IR, and fasting insulin, and were positively correlated with HDL-cholesterol, supporting previous reports on the association of NAFLD and serum ALT with low adiponectin levels in obese patients.^[42]^

Taken together, our findings in healthy individuals endorse epidemiological findings that elevated ALT values (including those within normal range) are strongly associated with NAFLD, insulin resistance, and probably, with an increased risk of T2DM.^[16]^

Elevated serum TNF-α levels have been described in patients with chronic liver disease of different etiologies,^[43,44]^ including NAFLD,^[45]^ and activation of the TNF-α system has been associated with insulin resistance^[46]^ and low adiponectin levels.^[45]^ In our study, serum TNF-α levels were higher in the individuals with NAFLD than in those without NAFLD, but TNF-α did not correlate with ALT, HOMA-IR, adiponectin, or any other parameter, in agreement with previous reports in obese patients.^[39]^ These findings might indicate that the TNF-α system is activated in NAFLD, but its relationship with insulin resistance and other metabolic risk factors remains to be elucidated.

It is well-documented that serum ALT levels cannot be used to predict nonalcoholic steatohepatitis or to differentiate between simple steatosis and nonalcoholic steatohepatitis.^[13,23]^ A recent report that used ^1^H-MRS to quantify liver fat content, and studied liver biopsies to assess the severity of liver disease in NAFLD patients found that elevated serum ALT was strongly associated with liver fat content, but not with inflammation, hepatocyte ballooning, or liver fibrosis.^[13]^ It has therefore been proposed that serum ALT might be a good indicator of NAFLD^[13]^ and a predictor of cardio-metabolic disorders, regardless of the possible progression to steatohepatitis or liver cirrhosis.^[17,20]^ Unfortunately, most studies on the sensitivity and specificity of serum ALT as a biomarker of NAFLD were aimed at identifying nonalcoholic steatohepatitis,^[23,47]^ whereas others exclusively used abdominal ultrasound to assess liver fat content,^[25,26,48]^ an operator-dependent method that offers inadequate sensitivity and specificity in cases of mild and moderate NAFLD and more importantly, does not provide a quantification of liver triglyceride content.^[29,31]^ Likewise, indexes developed to detect NAFLD have not proven useful for routine clinical practice in the general population due to their high complexity.^[49]^ Our study provides the first report on the diagnostic accuracy, sensitivity, and specificity of serum ALT as a biomarker of NAFLD in healthy individuals from the general population using ^1^H-MRS for liver triglyceride quantification. In our study, in addition to the strong correlation between serum ALT and liver triglyceride content, regression analysis showed that serum ALT was the main predictor of NAFLD even after adjustment for sex, age, BMI, and waist circumference. Furthermore, the ROC curve yielded an AUC of 0.93 and showed that serum ALT value ≥23 IU/L predicted the presence of NAFLD, with a sensitivity of 0.94 and specificity of 0.72. We found that 48% of individuals with serum ALT below 40 IU/L had NAFLD, and these individuals had significantly elevated HOMA-IR and lower serum levels of adiponectin and HDL-cholesterol with respect to those with serum ALT below 23 IU/L. These findings support proposals to reduce the threshold for “normal” serum ALT.^[17,21]^ Only 10% of participants with ALT below 23 IU/L had NAFLD, suggesting that this threshold would identify the large majority of individuals with hepatic steatosis. The detection of NAFLD is important to allow clinical counseling on the risk of insulin resistance, T2DM, and coronary heart disease.

The major strengths of this study are that ^1^H-MRS was used to quantify liver triglyceride content, and study subjects met strict exclusion criteria. In addition, the anthropometric, biochemical, and ^1^H-MRS measurements were performed within a 24-hour period.

Limitations of this study include the relatively high proportion of participants with NAFLD, possibly because most individuals in the eligible population had normal blood analyses and may therefore have been less willing to participate in comparison with those with elevated ALT values. We also acknowledge that this was a cross-sectional study based on biochemical determinations at a single time point in each participant. It proved possible to classify participants into different groups with adequate statistical power, but the sample size was relatively small, and further studies in wider samples and with an independent validation cohort are required to verify our findings and to establish how well these results apply to a different subset of patients.

## 5 Conclusions

In conclusion, this study shows that serum ALT is a sensitive, simple, and reliable biomarker of NAFLD if the normal ALT value is revised and established at a lower level. Thus, in the present series of healthy individuals, an ALT threshold of 23 IU/L identified 94% of participants with NAFLD, who might be more at risk of cardiometabolic events, allowing the appropriate clinical counseling to the identified individuals. The low cost and wide availability of this screening test facilitate its routine application in primary care.
